# Effects of High Moisture Corn Feeding on Weight Performance, Serum Immune Indices, Rumen Fermentation, and Metabolomics in Kazakh Rams

**DOI:** 10.3390/ani16071030

**Published:** 2026-03-27

**Authors:** Buweiaizhaer Maimaitimin, Tong Li, Subinuer Abuduli, Kadeliya Abudureyimu, Linhai Song, Liang Yang, Wei Shao, Wanping Ren

**Affiliations:** College of Animal Science, Xinjiang Agricultural University, Urumqi 830000, China; 17590513307@163.com (B.M.); 13853415007@163.com (T.L.); 13649970090@163.com (S.A.); 13699915053@163.com (K.A.); 15009360416@163.com (L.S.); yangliangagu@sina.com (L.Y.); dksw@xjau.edu.cn (W.S.)

**Keywords:** high-moisture corn, Kazakh rams, weight performance, immune indices, microbial diversity, rumen metabolomics

## Abstract

This study aimed to investigate the effects of feeding high-moisture corn (HMC) on weight performance, serum immune and antioxidant indices, rumen fermentation, microbial community, and metabolomics in Kazakh rams. A total of 32 healthy Kazakh rams were randomly divided into a control group (CT, diet with only ordinary crushed corn) and an experimental group (GS, diet with 50% ordinary crushed corn + 50% HMC), following a 7-day adaptation period and a 120-day trial period. Results showed that the F/G was significantly lower in the GS group than in the CT group (*p* < 0.05). FBW, net weight gain and ADG increased by 4.58%, 8.69%, and 8.70%, respectively, while ADFI decreased by 7.04% (*p* > 0.05). Regarding serum immune indices, IgA in the GS group was significantly higher at 40 d (*p* < 0.01), and IgM was significantly higher at 40, 80, and 120 d (*p* < 0.05). For antioxidant indices, the SOD activity in the GS group was significantly higher than that in the CT group at 120 d (*p* < 0.01). The CAT activity in the GS group was significantly higher at 40, 80, and 120 d (*p* < 0.01). Among rumen fermentation parameters, the concentration of butyric acid in the GS group was significantly lower than in the CT group (*p* < 0.01). Microbial diversity analysis indicated no significant differences in Alpha- and Beta-diversity of rumen microorganisms between the two groups. However, the relative abundance of *Firmicutes_A* at the phylum level was significantly higher in the GS group (*p* < 0.05), and the abundance of *Cryptobacteroides* was significantly higher than in the CT group (*p* < 0.01). Rumen metabolomic analysis identified a total of 1357 differential metabolites, among which 1130 showed significant differences, with 459 upregulated and 671 downregulated. These were mainly enriched in pathways such as Glutathione metabolism, Beta-alanine metabolism, Sphingolipid metabolism, and lysine degradation. In conclusion, feeding HMC can improve feed conversion efficiency and weight performance in Kazakh rams, regulate the structure of dominant rumen microorganisms, and enhance immune and antioxidant capacities.

## Simple Summary

Feed costs represent a major expense in sheep production, creating demand for cost-effective feeding strategies that maintain animal health and performance. This study evaluated whether replacing a portion of ordinary crushed corn with HMC could improve growth performance and physiological status in Kazakh rams, a prominent breed in northwest China. A total of 32 animals were assigned to either a conventional diet or one containing 50% HMC for 120 days. Rams receiving HMC demonstrated superior feed conversion efficiency and enhanced weight gain. Immunological assessment revealed elevated serum immunoglobulins, while antioxidant enzyme activities were significantly increased, indicating improved immune function and oxidative stress defense. Rumen fluid analysis showed modified fermentation patterns and beneficial shifts in microbial community structure, alongside metabolic changes linked to enhanced antioxidant capacity. These findings demonstrate that incorporating HMC into sheep diets can improve production efficiency and physiological health, offering a practical nutritional strategy for sustainable livestock farming.

## 1. Introduction

Corn is a widely used energy source in meat sheep diets, and its price significantly impacts the economic efficiency of sheep farming. Improving the utilization efficiency of corn, reducing feed costs, and enhancing production efficiency has become an urgent need to promote cost reduction, efficiency improvement, and sustainable development in the sheep industry. Ordinary crushed corn is typically harvested when the corn is fully mature and the kernel moisture content has dropped below 14%. It primarily relies on drying to reduce moisture for extended preservation; however, if storage conditions are too humid or improperly managed, it is prone to mold and spoilage. In addition, this processing method requires field drying or mechanical drying to achieve a safe moisture level, and the fuel and equipment costs associated with the drying process significantly drive up feed expenses [1]. In contrast, high-moisture corn (HMC) is a high-energy feed produced by harvesting corn kernels at 25–38% moisture content, followed by crushing, processing, and sealed storage for fermentation. Depending on the raw material, HMC can be classified into types such as grain silage and whole ear silage with corn cobs. The harvesting timing is critical for quality, with the optimal period being from late milk ripening to wax ripening when the grain moisture content reaches 28–42%. The processing technology primarily includes crushing to disrupt the grain structure, adding organic acids and lactic acid bacteria as silage fermentation agents to promote anaerobic fermentation, and compacting for sealed storage. In terms of nutritional properties, HMC effectively preserves nutrients, improves starch digestibility, enhances palatability, inhibits mold growth, reduces toxin risks, and its high fiber content helps maintain rumen health while reducing breeding costs. It is increasingly valued in the field of ruminant nutrition and feed science [2]. Studies have shown that compared to ordinary crushed corn, HMC improves starch digestibility, which is beneficial for enhancing energy utilization efficiency in ruminants, increasing microbial protein yield, improving ammonia nitrogen utilization, and thereby improving ruminant production performance [3]. Wet storage of HMC leads to the production of organic acids, including lactic acid and acetic acid. These acids lower the pH of the storage environment, inhibit bacterial growth, prevent mold proliferation, and thus reduce nutrient loss [4]. Application of HMC in dairy cow production can improve their performance, positively affecting nutrient digestibility, feed conversion rate, and milk yield [5]; replacing dry matter such as steam-flaked corn in the diet with HMC for dairy cows significantly reduced the production cost per kilogram of milk by CNY 0.12 [6]. Application of HMC in beef cattle production can improve performance such as feed intake, daily gain, and dry matter digestibility, effectively reducing feeding costs [7]; feeding HMC to beef cattle found that it could improve growth performance and carcass characteristics [8]; feeding HMC can increase the daily gain of lambs and reduce intramuscular cholesterol content [9]. Although HMC has been widely used in dairy and beef cattle feeding both domestically and internationally, research related to meat sheep remains relatively limited. Studies on the regulatory mechanisms of feeding HMC on rumen microbial diversity and metabolism in meat sheep have not been reported and require further in-depth research. Kazakh sheep are one of China’s three major coarse-wool sheep breeds, belonging to the meat-fat dual-purpose type of the primitive Xinjiang sheep lineage. They are widely raised in northwest China due to their characteristics of tolerance to roughage, excellent meat quality, and strong immunity [10], possessing broad development prospects. Based on this, the present study proposes the following hypotheses: Feeding HMC can improve the body weight performance and feed conversion efficiency of Kazakh rams by regulating ruminal fermentation patterns—moderately slowing starch degradation rates to maintain ruminal pH stability and increasing propionic acid proportion. Meanwhile, a stable ruminal environment helps reduce serum inflammatory markers and improve immune status. Based on ruminal metabolomics analysis, we expect differential metabolites to be primarily enriched in carbohydrate and energy metabolism pathways, thereby revealing the molecular mechanisms by which HMC affects production performance and immune function at the metabolic level. This study aims to elucidate the metabolic regulatory mechanisms by which high-moisture corn influences production performance and ruminal function in meat sheep, providing theoretical basis and practical guidance for the scientific application of HMC in efficient fattening production of meat sheep.

## 2. Materials and Methods

This experiment was conducted from October 2024 to March 2025 at a sheep farm in Wugongtai Town, Hutubi County, Xinjiang. The animal care and experimental procedures involved in this trial were approved by the Animal Welfare Ethics Committee of Xinjiang Agricultural University (Approval No: 20240806, Date of Issue: 19 February 2024).

### 2.1. Animal Management and Feeding

Thirty-two healthy Kazakh rams, approximately 6 months old and with similar body weights (35.47 ± 1.46 kg), were selected as experimental subjects. The experimental sheep were randomly divided into two groups: the control group (CT group) and the experimental group (GS group), with 16 sheep in each group (*p* > 0.05). The control group was fed with ordinary crushed corn, while the experimental group was fed with a mixed diet of 50% ordinary crushed corn and 50% HMC. Each group had 4 replicates, with 4 sheep per replicate, and the sheep within the same replicate were housed in the same pen. The HMC and ordinary crushed corn used in the experiment were both purchased from Xinjiang Tianrun Dairy Co., Ltd. (Urumqi, China). HMC fermentation employs a mixed feed additive (Shanghai Yuanyao Biological Co., Ltd., Shanghai, China), with the primary microbial community consisting of Lactobacillus (*Lactococcus lactis*, *Lactobacillus brevis*, 1.3 × 10^11^ cfu/g). The harvested HMC is crushed, inoculated with additives, and stored as wrapped silage in a cool and well-ventilated place.

The basic diet formula was designed according to the standard “Nutritional Requirements for Meat Sheep” (NY/T 816-2021) [11], with detailed composition shown in Table 1. The roughage portion of the diet consisted of cotton residue and whole plant corn silage. The experimental rams were uniformly dewormed and rumen function adjusted before the experiment began. The pre-experiment period lasted 7 days, and the main feeding period was 120 days. During the experiment, feeding was scheduled at 07:00 and 19:00 daily, with free drinking water. Feed intake was recorded per replicate and calculated as the difference between daily feeding and leftover feed. All experimental sheep were raised under identical environmental conditions to ensure comparability of the experimental results. The nutritional components of the ordinary crushed corn and HMC used in the diets are shown in Table 2.

### 2.2. Sample Collection and Testing

#### 2.2.1. Growth Performance

On day 0 and day 120 of the trial, all experimental sheep were weighed before morning feeding using an electronic scale (model DTC001-A, Dongmei, Shenzhen, China; accuracy: ±0.05 kg) in a fasted state. Each sheep was weighed three times consecutively, and the average was calculated, recorded as initial body weight (IBW) and final body weight (FBW), respectively. Average daily gain (ADG) was also calculated using the formula below:

ADG (kg/d) = (FBW (kg) − IBW (kg))/Trial days (d)

During the trial period, daily feed intake was recorded (weighed before feeding each day, and leftovers were weighed before feeding at 07:00 the next morning) to calculate the average daily feed intake (ADFI) and feed-to-gain ratio (F/G) for the entire period. The formulas are as follows:

ADFI (kg/d) = (Total feed offered (kg) − Total feed leftover (kg))/Trial days (d)

F/G = Dry matter intake (kg)/ADG (kg)

#### 2.2.2. Blood Sample Collection and Analysis

To assess immune status and antioxidant capacity, blood samples were obtained from all rams at four time points (days 0, 40, 80, and 120). Sampling was performed via jugular venipuncture prior to morning feeding after an overnight fast. Approximately 5 mL of blood was collected from each animal, Stand still at room temperature, and then centrifuged at 3000× *g* for 15 min to separate serum. The resulting supernatants were transferred into sterile 1.5 mL microcentrifuge tubes and stored at −80 °C until analysis. Serum concentrations of immunoglobulins (IgA, IgG, and IgM) were quantified using an automated biochemical analyzer (Mindray BS-420, Shenzhen, China) following the manufacturer’s protocols. Cytokines (IL-1β and TNF-α) and oxidative stress markers (SOD, CAT, MDA) were measured using commercial enzyme-linked immunosorbent assay (ELISA) kits (Beijing Huaying Biotechnology Co., Ltd., Beijing, China). All assays were performed according to the manufacturer’s instructions, and absorbance readings were recorded at the corresponding wavelengths (340 nm for IgA and IgM, 610 nm for IgG, 405 nm for SOD and CAT, 532 nm for MDA, and 450 nm for IL-1β and TNF-α) using a microplate reade (DR-200BS, Wuxi Huawei Delang Instrument Co., Ltd., Wuxi, China)

#### 2.2.3. Rumen Fluid Collection

On day 120 of the experiment, oral intubation sampling was performed using the GCYQ-1-A rumen fluid sampler (Shanghai Yifan Biotechnology Co., Ltd., Shanghai, China) 3 h after morning feeding. The initial 60 mL of effluent, was discarded, and a subsequent 60 mL of rumen fluid was collected. The sample was immediately filtered through four layers of sterile gauze, and the pH value was determined on-site using a portable pH meter (model 8601, AZ Instrument Corp., Shanghai, China). Following measurement, the rumen fluid was aliquoted into cryotubes and rapidly transferred to a liquid nitrogen tank for long-term storage prior to further analysis. All procedures were conducted in strict compliance with the Laboratory Biosafety General Requirements (GB 19489-2008) [13].

#### 2.2.4. Rumen Volatile Fatty Acid Analysis

After the rumen fluid collection was completed, the rumen fluid samples were sent to Novogene Bioinformatics Technology Co., Ltd. (Beijing, China) for testing. To determine rumen volatile fatty acids (VFA), 20 μL of the sample was added to the extraction reagent during pretreatment, and a series of concentration standards and isotope internal standards (e.g., acetic acid-D4) were prepared for quantitative calibration. The ultra-high performance liquid chromatography-tandem mass spectrometry (UHPLC-MS/MS) system was used, equipped with a Waters ACQUITY UPLC BEH C18 column (1.7 μm, 2.1 × 100 mm). The mobile phase consisted of a 10 mM ammonium acetate aqueous solution and a 50% acetonitrile-isopropanol mixture, with a column temperature of 40 °C and a flow rate of 0.3 mL/min. Target compounds were separated by gradient elution. The mass spectrometry employed electrospray ionization (ESI) in negative ion mode and multiple reaction monitoring (MRM) scanning to obtain peak area data for each VFA (including acetic acid, propionic acid, butyric acid, isobutyric acid, valeric acid, isovaleric acid, etc.). When establishing the standard curve, the concentration ratio of the standard to the internal standard was plotted on the x-axis, and the peak area ratio on the y-axis, with a correlation coefficient (r) > 0.99. The limit of quantification (LOQ) was determined based on the signal-to-noise ratio (S/N = 10). Method validation covered precision (intra-day and inter-day RSD ≤ 15%), accuracy (recovery rate 85–115%), and stability (RSD ≤ 15% after 24 h at 4 °C). After normalization, the quantitative results were further analyzed using multivariate statistical methods such as principal component analysis (PCA), cluster analysis, and correlation analysis to identify metabolic patterns. Differentially expressed metabolites were selected based on fold change (FC > 1.2 or <0.833) combined with t-test (*p*-value < 0.05). Finally, the potential of these metabolites as biomarkers was evaluated using ROC curves.

#### 2.2.5. Rumen Microbiota Analysis

Following rumen fluid collection, samples from each group were sent to Kaitai Biotechnology Co., Ltd. (Shanghai, China) for microbiome analysis. Microbial diversity was assessed by amplifying the hypervariable V3-V4 region of the bacterial 16S rRNA gene using the barcode-specific primers 338F (5′-ACTCCTACGGGAGGCAGCA-3′) and 806R (5′-GGACTACHVGGGTWTCTAAT-3′) with NEB Q5 High-Fidelity DNA Polymerase. The amplification protocol consisted of an initial denaturation at 98 °C for 30 s, followed by 25–27 cycles of denaturation at 98 °C for 15 s, annealing at 50 °C for 30 s, and extension at 72 °C for 30 s, with a final extension at 72 °C for 5 min. PCR products were quantified using the Quant-iT PicoGreen dsDNA Assay Kit on a microplate reader (BioTek, FLx800, USA) and pooled in equimolar ratios based on the required sequencing depth for each sample. Sequencing libraries were constructed using the Illumina TruSeq Nano DNA LT Library Prep Kit, which included DNA end repair, 3′ adenylation, adapter ligation with index sequences, and library amplification. Purification at different stages was performed using BECKMAN AMPure XP beads. The final library quality was assessed on a LabChip system, and libraries passing quality control were sequenced on an Illumina NovaSeq 6000 platform using the NovaSeq 6000 SP Reagent Kit (500 cycles), generating 2 × 250 bp paired-end reads. Raw sequencing data were processed using QIIME2 (2023.7). The DADA2 plugin was employed for quality filtering, denoising, merging of paired-end reads, and removal of chimeric sequences to generate amplicon sequence variants (ASVs). Taxonomic assignment of ASVs was performed using a pretrained Naive Bayes classifier based on the Greengenes2 reference database. Alpha diversity metrics, including Chao1, Observed species, Shannon, Simpson, Faith’s PD, Pielou’s evenness, and Good’s coverage, were calculated using the diversity plugin after rarefying samples to an even sequencing depth. Beta diversity was assessed using principal coordinate analysis (PCoA) based on Bray–Curtis dissimilarity, Jaccard distance, unweighted UniFrac, and weighted UniFrac distances. Permutational multivariate analysis of variance (PERMANOVA) was used to test for significant differences in microbial community structure between groups. Linear discriminant analysis effect size (LEfSe) was performed to identify differentially abundant taxa between groups, with an LDA score threshold of >2.0. Co-occurrence network analysis was conducted based on Spearman’s correlation coefficients (|r| > 0.8, *p* < 0.01) to explore microbial interactions. Functional potential of the microbial community was predicted using PICRUSt2, with KEGG pathway enrichment analysis performed to identify differentially enriched metabolic pathways between groups.

#### 2.2.6. Rumen Metabolomics

After completing the rumen fluid collection, the samples were sent to Novogen Bioinformatics Technology Co., Ltd. (Beijing, China) for liquid chromatography-mass spectrometry (LC-MS) analysis. The study first performed sample pretreatment, extracting metabolites from the rumen fluid samples, and set up QC samples (made by mixing all experimental samples in equal volumes) to monitor instrument stability. The detection was performed using liquid chromatography-mass spectrometry (LC-MS) technology, with the instrument platform being the Q Exactive™ HF-X high-resolution mass spectrometer. The raw data from the instrument were converted to mzXML format using the ProteoWizard software, (3.0.x) followed by peak extraction, peak alignment, and retention time correction using the XCMS software (4.8.0), and the total peak area extracted from the samples was standardized. Metabolite identification was completed by searching high-quality secondary spectrum databases, and metabolites with a coefficient of variation (CV) less than 30% in the QC samples were retained as the final identification results. Data quality control included correlation analysis of QC samples (Pearson correlation coefficient) and principal component analysis (PCA) of the total samples to assess the stability of the detection process and data quality. Subsequently, multivariate statistical analyses were performed, including principal component analysis (PCA) to observe the overall distribution trends between groups, and partial least squares discriminant analysis (PLS-DA) to establish classification models, which were evaluated for reliability through 7-fold cross-validation and permutation testing. The screening of differential metabolites was performed using the thresholds of VIP > 1.0, FC > 1.5, or FC < 0.667 with a *p*-value < 0.05. Subsequent analyses were performed on the selected differential metabolites, including cluster analysis, K-Means clustering, correlation analysis, and Z-score analysis. Pathway annotation and enrichment analysis were conducted using the KEGG database (hypergeometric test, with a *p*-value <0.05 as the significant enrichment threshold). GSEA analysis was also performed to address the limitations of traditional enrichment analysis. Finally, ROC curve analysis was employed to evaluate the predictive capacity of differential metabolites as potential biomarkers (the closer the AUC value is to 1, the higher the predictive accuracy).

### 2.3. Data Analysis

In this experiment, growth performance parameters, including ADF, ADG, and F/G, were evaluated based on feed intake recorded at the pen level, with the pen-level averages used directly for statistical analysis. Similarly, feed intake for serum immunity, antioxidant indicators, and rumen fermentation parameters was also recorded at the pen level, and the pen-level means were directly analyzed. For microbial diversity and metabolome analysis, rumen fluid samples from two rams randomly selected from each pen were used to obtain the average value per pen prior to analysis. All data were analyzed using SPSS 27.0 statistical software (IBM Corporation, Armonk, NY, USA). Independent sample t-tests were used to compare the CT group and the GS group, with pen as the experimental unit. Results are expressed as mean ± standard deviation (SD). The statistical significance level was set at *p* < 0.05, and extremely significant differences were set at *p* < 0.01.

## 3. Results

### 3.1. Effects of Feeding HMC on Weight Performance of Kazakh Rams

Table 3 shows the effects of feeding HMC on the weight performance of Kazakh rams. The data showed that the FBW, net weight gain and ADG of the GS group were 4.58%, 8.69%, and 8.70% higher than those of the CT group respectively, and the ADFI was 7.04% lower than that of the CT group (*p* > 0.05). The F/G of the GS group was significantly lower than that of the CT group (17.28%), and the differences were statistically significant (*p* < 0.05).

### 3.2. Effects of Feeding HMC on Blood Indices of Kazakh Rams

#### 3.2.1. Effects of Feeding HMC on Serum Immune Indices of Kazakh Rams

Table 4 shows the effects of feeding HMC on serum immune indices of Kazakh rams. At 40 d, IgA content in the GS group was significantly higher than in the CT group (*p* < 0.01). At 40 d, 80 d, and 120 d, IgM content in the GS group was significantly higher than in the CT group (*p* < 0.05). There were no significant differences in IgG, IL-1β, TNF-α, and other indices between the two groups at any time point (*p* > 0.05).

#### 3.2.2. Effects of Feeding HMC on Serum Antioxidant Indices of Kazakh Rams

Table 5 shows the effects of feeding HMC on serum antioxidant indices of Kazakh rams. At 120 d, SOD levels in the GS group were significantly higher than in the CT group (*p* < 0.01). At 40 d, 80 d, and 120 d, CAT levels in the GS group were significantly higher than in the CT group (*p* < 0.01). There were no significant differences in MDA and T-AOC indices between the two groups at any time point (*p* > 0.05).

### 3.3. Effects of Feeding HMC on Rumen Fluid Fermentation and Microbial Diversity in Kazakh Rams

#### 3.3.1. Effects of HMC on Rumen Fluid Fermentation in Kazakh Rams

Table 6 shows the effects of feeding HMC on rumen fluid fermentation indices of Kazakh rams. The data show that butyric acid concentration in the CT group was significantly higher than in the GS group (*p* < 0.01). Concentrations of 2-methylbutyrate and valeric acid in the GS group were significantly higher than in the CT group (*p* < 0.05). Other indices such as pH, acetic acid, propionic acid showed no significant differences (*p* > 0.05).

#### 3.3.2. Effects of Feeding HMC on Alpha and Beta Diversity Analysis of Rumen Fluid Microorganisms in Kazakh Rams

##### Alpha Diversity Analysis

**Figure 1:**
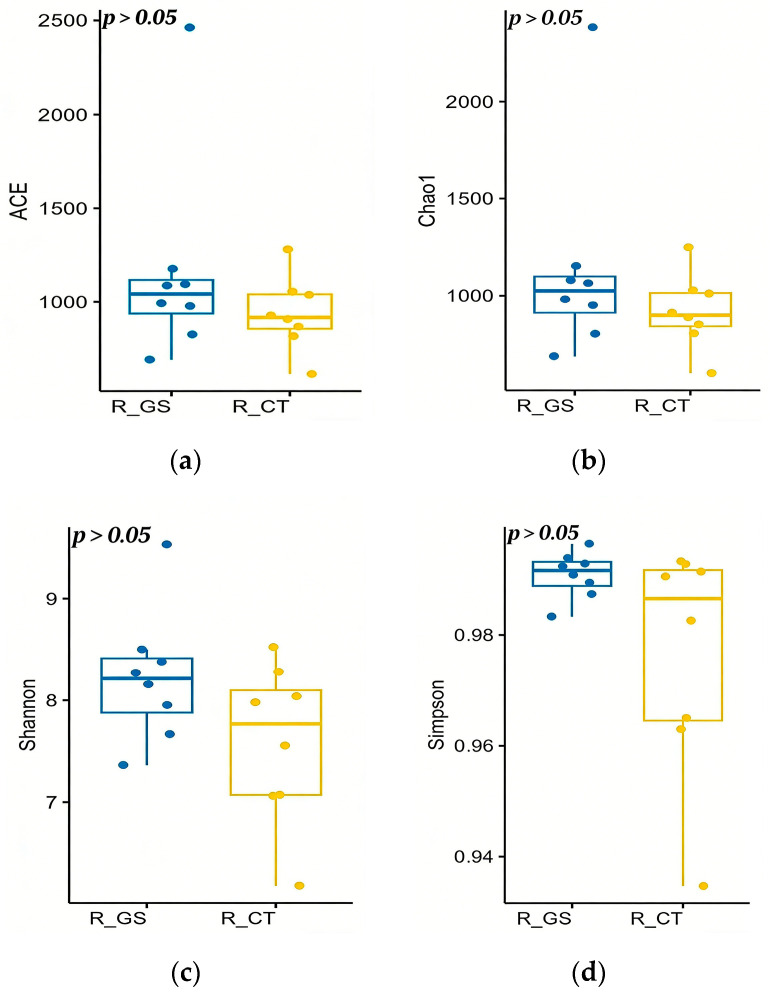
Effects of feeding HMC on Alpha diversity of rumen fluid microorganisms in Kazakh rams. (**a**) ACE (Abundance-based Coverage Estimator) is an index used to estimate the number of species in a community. (**b**) Chao1 index: Estimates the total number of species in a community, primarily reflecting species’ richness. A higher value indicates greater species richness and a larger potential number of undiscovered species. (**c**) Shannon index: Incorporates both species richness and the evenness of species abundance. A higher value indicates greater species richness, more uniform distribution of individuals, higher uncertainty, and thus greater overall diversity. (**d**) Simpson index: comprehensively reflects both species’ richness and evenness. A value closer to 1 suggests not only higher species richness but also a more uniform distribution of individuals among species, with no single dominant species.

The effects of feeding HMC on the Alpha diversity of the rumen fluid microbial community are shown in Figure 1. Replacing ordinary crushed corn with HMC in the diet showed no significant differences in ACE, Chao1, Shannon, and Simpson indices of the rumen fluid in Kazakh rams (*p* > 0.05).

##### Beta Diversity Analysis

**Figure 2:**
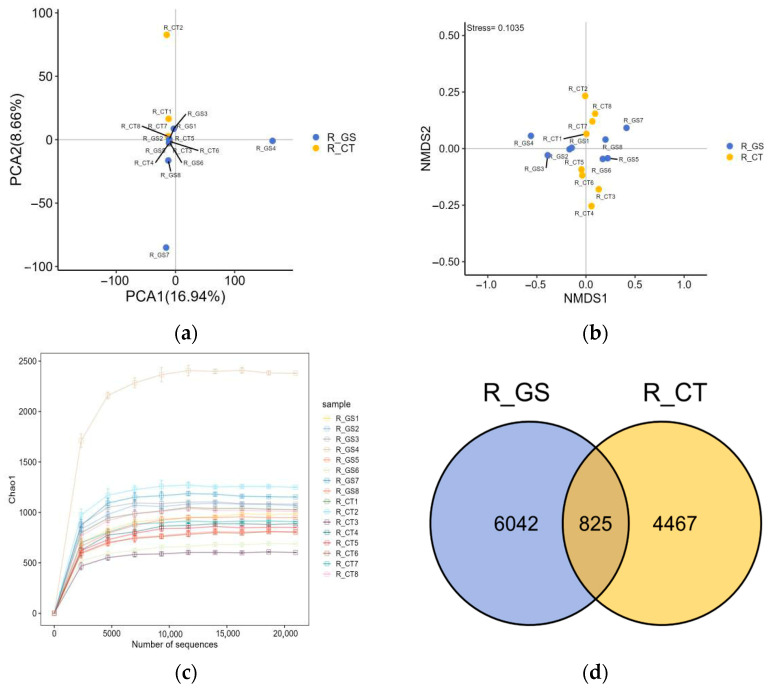
Effects of feeding HMC on Beta diversity of rumen fluid microorganisms in Kazakh rams. (**a**) Principal Coordinates Analysis (PCoA) of Beta diversity (**b**) Non-metric Multidimensional Scaling (NMDS) of Beta diversity; (**c**) Rarefaction curves; (**d**) Venn diagram, OTUs, operational taxonomic units.

The treatment groups were evaluated using principal component analysis (PCoA) and NMDS. The results clearly showed significant overlap in the microbial communities of the two treatment groups, as shown in Figure 2a,b. Analysis of beta diversity of rumen fluid indicated that the microbial communities of the GS and CT groups exhibited similar characteristics, with no significant differences found. The Chao1 index rarefaction curve for rumen fluid samples (Figure 2c) showed a gentle trend, indicating that sequencing depth was nearing saturation. This suggests that the sequencing depth was reliable, able to capture a substantial portion of the microorganisms in the samples, allowing for accurate analysis of their composition and ensuring sufficient coverage for microbial diversity analysis. Based on the optimized sequences, a total of 11,334 OTUs were obtained from both groups, with 825 OTUs present in all samples (Figure 2d).

#### 3.3.3. Effects of Feeding HMC on Species Distribution Analysis at Phylum and Genus Levels in Rumen Fluid of Kazakh Rams

**Figure 3:**
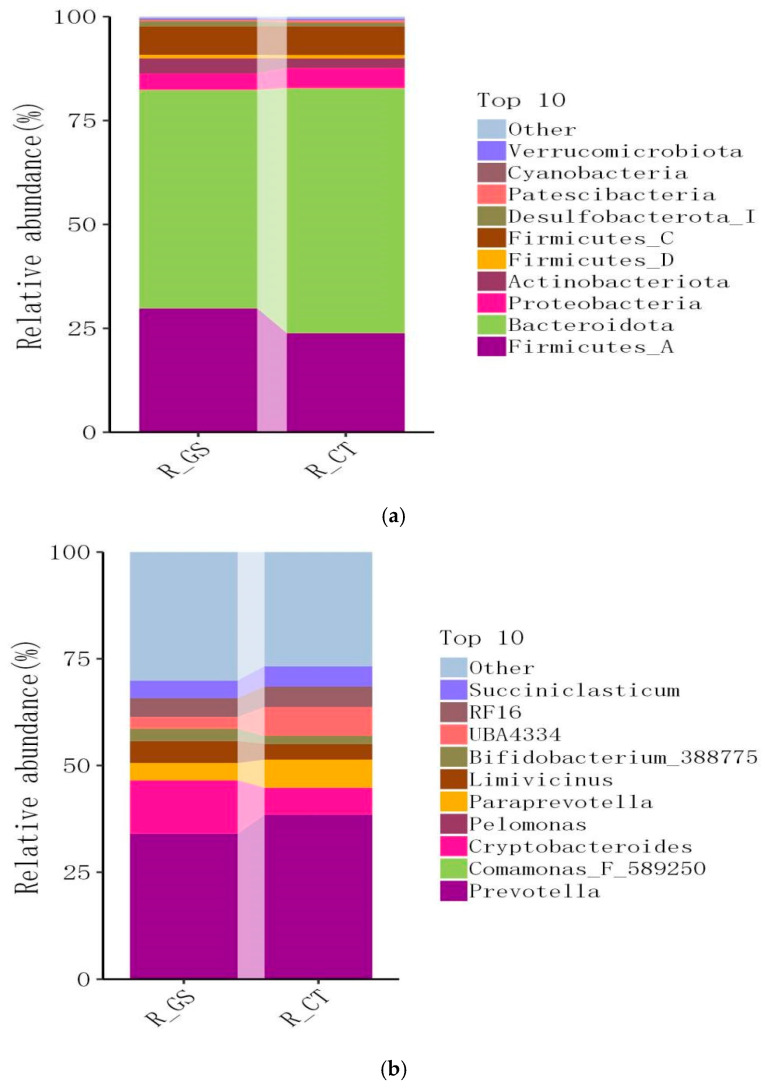
Effects of feeding HMC on the relative abundance of microbial communities at the (**a**) phylum and (**b**) genus levels in the rumen fluid of Kazakh rams.

A total of 24 microbial phyla were identified in this study, with *Bacteroidota*, *Firmicutes_A*, and *Firmicutes_C* dominating across all classifications (Figure 3a). At the genus level, 338 microbial genera were identified in the samples, with *Prevotella*, *Cryptobacteroides*, and *Succiniclasticum* being the most prominent (Figure 3b). At the phylum level, the abundance of *Firmicutes_A* in the GS group was significantly higher than in the CT group (*p* < 0.05). At the genus level, the abundance of *Cryptobacteroides* in the GS group was significantly higher than in the CT group (*p* < 0.01).

#### 3.3.4. LEfSe Analysis of Rumen Fluid Microorganisms

**Figure 4:**
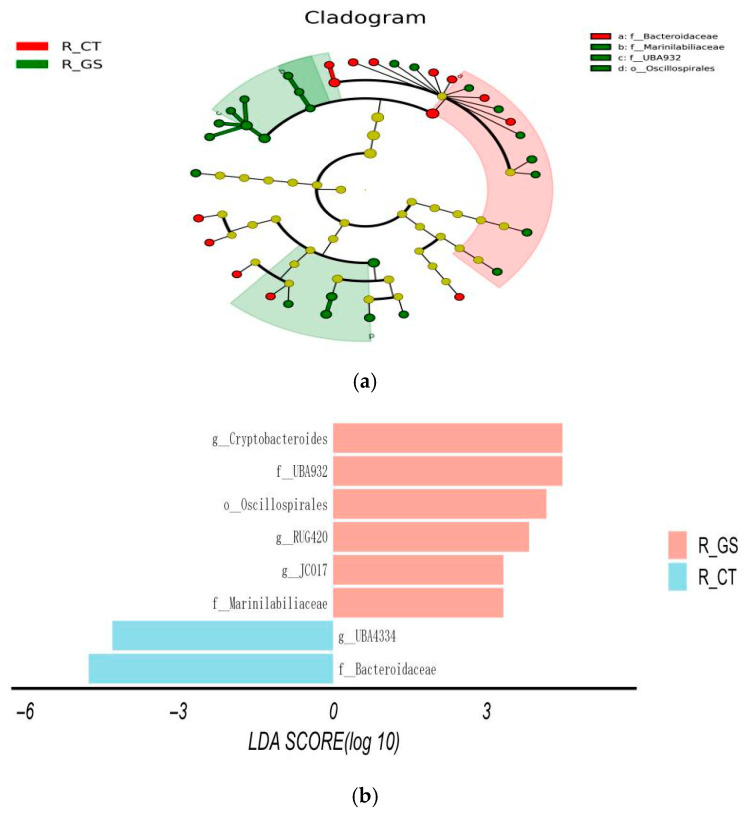
LEfSe analysis of the rumen fluid microbial community in Kazakh rams fed HMC. (**a**) Cladogram showing the phylogenetic distribution of differentially abundant taxa between the CT and GS groups. Circles from the inner to the outer rings represent taxonomic levels from phylum to genus. Each circle’s diameter is proportional to the relative abundance of the taxon. Red indicates taxa enriched in the GS group, green indicates taxa enriched in the CT group, and yellow indicates taxa with no significant difference.; (**b**) Histogram of Linear Discriminant Analysis (LDA) scoresfor differentially abundant taxa (LDA score > 2.0).

Figure 4a,b show the significant differences in rumen fluid microbial taxa between the two groups. In the GS group, the abundance of six bacterial taxa was significantly higher than in the CT group (LDA value >2): *Cryptobacteroides*, *UBA932*, *Oscillospirales*, *RUG420*, *JC017*, and *Marinilabiliaceae* (*p* < 0.05). Conversely, the abundance of *Bacteroidaceae* and *UBA4334* in the GS group was significantly lower than in the CT group (*p* < 0.05).

### 3.4. Effects of Feeding HMC on Rumen Metabolomics in Kazakh Rams

#### 3.4.1. Principal Component Analysis

**Figure 5:**
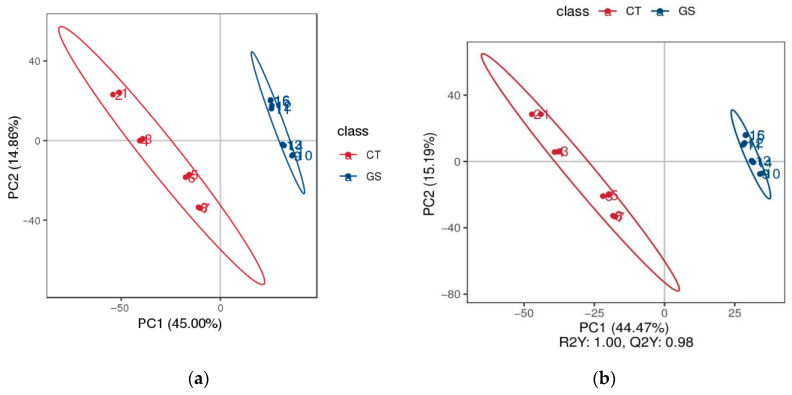
Principal Component Analysis (PCA) plot, PLS-DA score scatter plot, and permutation validation plot. (**a**) In the figure, the horizontal axis PC1 and vertical axis PC2 represent the scores of the first and second principal components, respectively. Scattered points of different colors indicate samples from different experimental groups, and ellipses denote the 95% confidence intervals (95% confidence ellipses cannot be displayed when the number of biological replicates is less than 4). (**b**) The scatter plot displays the scores of samples on the first principal component (horizontal axis) and the second principal component (vertical axis). R2Y indicates the model’s explanatory power, while Q2Y evaluates the predictive performance of the PLS-DA model. A higher R2Y than Q2Y signifies a well-established model. The rank test shows the correlation between the randomly grouped Y and the original grouped Y (horizontal axis), with the vertical axis representing the scores of R2 and Q2.

The results of principal component analysis are shown in Figure 5. The PCA score plot (PC1 vs. PC2) shows significant differences in the distribution of metabolites in rumen samples between the CT and GS groups of Kazakh rams in both positive and negative ion modes (Figure 5a). The orthogonal partial least squares-discriminant analysis (OPLS-DA) score plot clearly shows significant distinction and separation between the GS and CT groups (Figure 5b), indicating that HMC significantly affects the rumen metabolism of Kazakh rams.

#### 3.4.2. Screening and Analysis of Differential Metabolites

**Figure 6:**
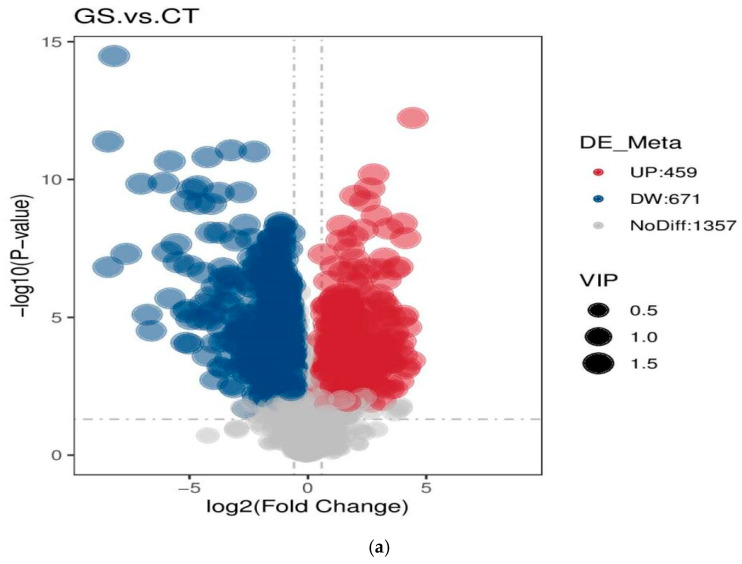

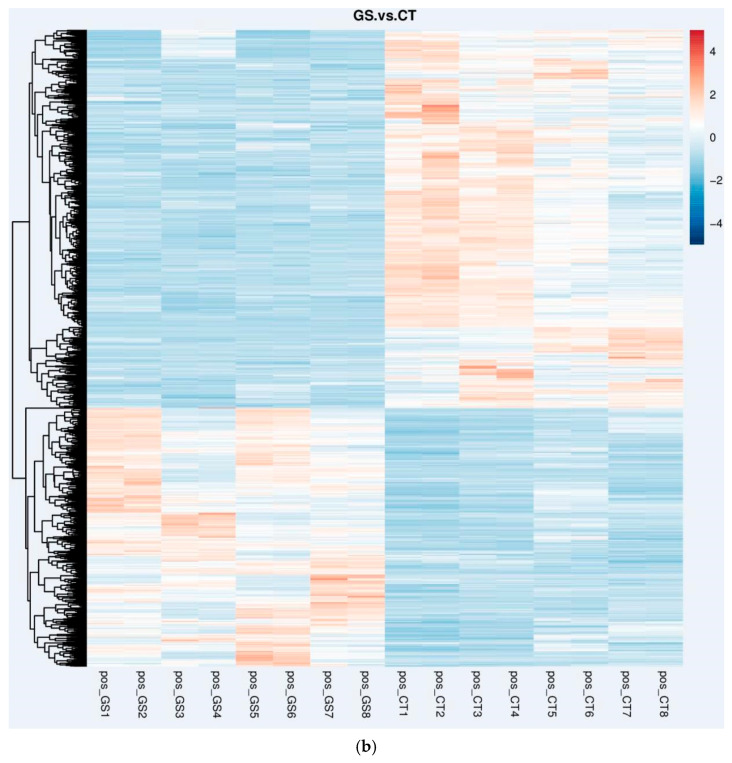
Volcano plot (**a**) and cluster heatmap (**b**) of differential metabolites in GS_vs._CT groups. (**a**) The volcano plot visually displays the overall distribution of differential metabolites. The horizontal coordinate represents the log2 fold change in metabolites between different groups, and the vertical coordinate represents the significance level (-log10(*p*-value)). Each point in the volcano plot represents a metabolite. Significantly upregulated metabolites are represented by red dots, and significantly downregulated metabolites are represented by blue dots. The size of the dot represents the VIP value. (**b**) Vertical clustering is for samples, and horizontal clustering is for metabolites. Shorter branches indicate higher similarity. Red represents upregulated differentially expressed metabolites, and blue represents downregulated differentially expressed metabolites.

The volcano plot visually shows the overall distribution of differential metabolites. The differential metabolite data from the two groups were normalized by unit variance scaling (UV), and then cluster heatmap analysis was performed on all samples (heatmap drawn using the R software (4.4.2) pheatmap package). A total of 1357 differential metabolites were detected between the two groups, among which 1130 were significantly different (VIP > 1, FC > 1.5 or FC < 0.667, *p* <0.05), 459 differential expressions were up-regulated, and 671 differential expressions were down-regulated (Figure 6a). According to the cluster heatmap of differential metabolites for CT vs. GS, the clustering effect of differential metabolites between the two groups was good (Figure 6b).

#### 3.4.3. KEGG Enrichment Analysis of Pathways for Differential Metabolites

**Figure 7:**
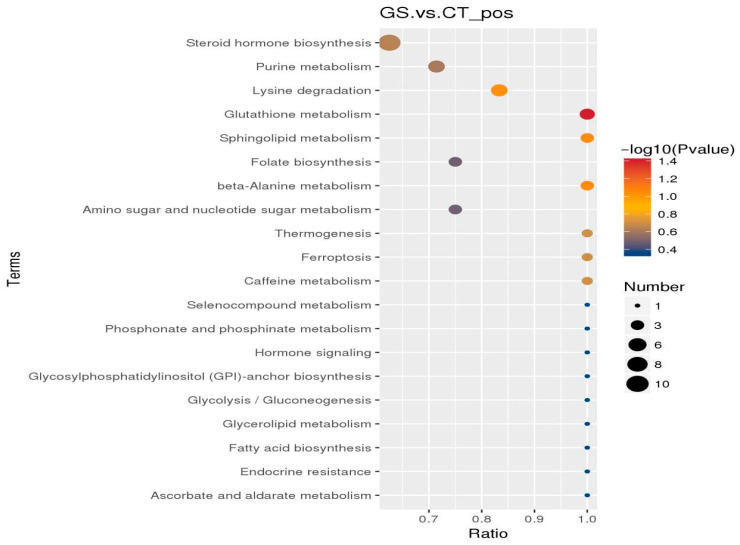
POS-KEGG enrichment impact factor bubble chart for differential metabolites in GS_vs._CT group. In the figure, the horizontal coordinate is x/y (number of differential metabolites in the corresponding metabolic pathway/total number of metabolites identified in that pathway). A larger value indicates a higher degree of enrichment of differential metabolites in that pathway. The color of the points represents the *p*-value from the hypergeometric test; a smaller value indicates greater reliability and statistical significance of the test. The size of the points represents the number of differential metabolites in the corresponding pathway; larger size indicates more differential metabolites within that pathway.

The KEGG enrichment analysis of differential metabolites (Figure 7) revealed that the differential metabolites in the rumen of two groups of rams were primarily enriched in 70 entries, with the top 20 entries displayed in the figure. Among these, the glutathione metabolism (GSH) pathway was significantly enriched (*p* < 0.05).

#### 3.4.4. Screening Results of Differential Metabolites

Table 7 shows the screening results of differential metabolites in the rumen of Kazakh rams fed HMC. KEGG pathway enrichment analysis showed that the GSH metabolic pathway was significantly different between the two groups (*p* = 0.03). In this pathway, compared with the CT group, the GS group identified a total of 4 significantly different metabolites: The contents of 5-oxoproline and dehydroascorbic acid were significantly increased (*p* < 0.01), while the contents of spermidine and cadaverine were significantly decreased (*p* < 0.05).

## 4. Discussion

### 4.1. Effects of Feeding HMC on Weight Performance of Kazakh Rams

Growth performance is a key trait affecting the economic efficiency of meat sheep [14]. The results of this experiment show that FBW, net weight gain, ADG, and ADFI of rams in the GS group were all improved, and the F/G was significantly improved. Stock, R. A et al. [15] showed that steers fed HMC had superior ADG and F/G compared to the ordinary crushed corn group; studies on beef cattle fattening also found that HMC had no significant effect on final weight and daily gain of experimental cattle, but F/G was lower than the control group [16], which is consistent with the results of this experiment. This indicates that using HMC as a replacement for ordinary crushed corn in the diet can improve the growth performance of Kazakh rams. This effect may be related to the silage characteristics of HMC. The ensiling process can reduce the loss of nutrients in corn kernels and disrupt the starch-protein complex structure [17], thereby maximizing the absorption and utilization of nutrients by the rumen after ingestion by the sheep and improving feed digestion efficiency. Therefore, in meat sheep production practice, rational application of HMC in the basal diet can help improve weight gain performance and feed conversion rate while reducing feeding costs.

### 4.2. Effects of Feeding HMC on Blood Indices of Kazakh Rams

Serum immunoglobulins are core indicators for evaluating the humoral immune level of animals, and their increased concentration usually reflects enhanced immune capacity [18]. The results of this experiment show that serum levels of IgA and IgM in Kazakh rams fed HMC were significantly increased at different time points, while IgG levels remained stable. J.Q. Wang et al. [19] found that supplementing HMC in weaned piglets significantly reduced the number of Escherichia coli in the intestines, thereby enhancing immune responses. Although the species are different, they reflect the potential impact of HMC on immunity; Wang Jinfei et al. [20] observed increased serum IgM and IgA content when adding 40% whole corn silage to the diet of ewe lambs, which is consistent with the results of this experiment. This may be related to organic acids such as lactic acid and acetic acid produced during the wet storage of HMC. These organic acids can inhibit the activity of harmful bacteria, promote the proliferation of beneficial bacteria, and stimulate antibody secretion in mesenteric lymph nodes, thereby enhancing the body’s immune capacity [21]. Furthermore, Pickworth C L et al. [22] pointed out that the fermentation process has a protective effect on beta-carotene in corn. HMC contains higher levels of beta-carotene than ordinary corn. Beta-carotene can be converted into vitamin A in the body, which has immunomodulatory functions [23]. Therefore, it is speculated that the improvement in immune function in Kazakh rams may also be related to the higher beta-carotene content in HMC. In summary, feeding HMC can significantly increase serum IgA and IgM levels in Kazakh rams, effectively enhancing the body’s immune capacity, and contributing to improved health and growth performance in ruminants.

Serum antioxidant indices are important markers reflecting animal metabolism and health status. SOD and CAT are key antioxidant enzymes in the body, capable of synergistically scavenging superoxide radicals and hydrogen peroxide to protect tissue cells from oxidative damage [24]. The results of this experiment show that feeding HMC significantly increased the activity of SOD and CAT in the serum of Kazakh rams, indicating that HMC can effectively enhance the antioxidant capacity of meat sheep. Shang Songlin et al. [25] reported that HMC could increase serum SOD activity in dairy cows, thereby enhancing their antioxidant function, which is consistent with the results of this experiment. In this experiment, CAT activity continued to increase significantly after feeding HMC, suggesting that HMC has a stable and lasting promoting effect on the key enzyme for hydrogen peroxide clearance. The increase in CAT activity not only helps alleviate oxidative stress and maintain intracellular redox homeostasis but may also support overall animal health, which could indirectly contribute to the improved feed efficiency observed in the GS group [26]. This may be related to the lactic acid bacteria enriched during the fermentation process of HMC [4]. Lactic acid bacteria have strong antioxidant capacity and can help the body cope with oxidative stress by secreting antioxidant enzymes and alcohols and other metabolites [27]. In conclusion, feeding fermented HMC can effectively increase the activity of SOD and CAT in the serum of Kazakh rams, enhance the body’s antioxidant capacity, and thereby promote animal health and growth performance.

### 4.3. Effects of Feeding HMC on Rumen Fluid Fermentation and Microbial Diversity in Kazakh Rams

The pH of rumen fluid is an indicator for assessing the acidity and alkalinity of the rumen internal environment in ruminants, typically ranging from 5.5 to 7.0. The composition and nutritional characteristics of the diet significantly influence rumen fermentation processes, playing a key role in shaping the rumen microbial ecosystem and its metabolic functions [28]. In this experiment, the rumen pH of rams in both groups was within the normal range, indicating that neither diet had a negative impact on rumen fermentation. However, the rumen fluid pH of rams in the GS group was higher than that of the control group. Peng Shuyu et al. [29] used different ratios of ordinary crushed corn and HMC for 48 h in vitro rumen fermentation and found that pH decreased with increasing HMC proportion; Coulson Caitlin A et al. [2] found that bulls fed HMC had lower pH compared to those fed dry corn, which is inconsistent with the results of this experiment. The reason may be that the starch digestibility of HMC in the rumen is higher than that of ordinary crushed corn [30], and the sampling time point in this trial was relatively late. The starch in HMC for the GS group rams was almost completely fermented in the rumen, thus affecting the measurement results of fermentation parameter-related indicators.

The concentration of VFAs in the rumen reflects the metabolic activity of rumen microorganisms on dietary carbohydrates [31]. After VFAs are produced by rumen microorganisms fermenting the diet, they are absorbed through the rumen wall into the bloodstream, providing energy for the body or being synthesized into fat [32]. In this experiment, the content of acetic acid, propionic acid, and total VFAs in the experimental group was lower than in the control group. The reason may be that when HMC enters the rumen, it is quickly decomposed by rumen microorganisms to produce VFAs, which are then directly absorbed by the rumen wall, resulting in lower VFA content in the rumen fluid of the experimental rams.

The taxonomic richness and community structure of the rumen microbiome are key factors regulating rumen function, and their composition is primarily influenced by diet. In this study, dietary addition of HMC did not significantly change the diversity and composition of rumen fluid microorganisms, which is consistent with the findings of Bach A et al. [33].

The animal gastrointestinal tract is a complex ecosystem of symbiotic interactions between the host and microorganisms. Beneficial bacteria play key roles in inhibiting pathogen proliferation, regulating immunity, promoting fiber degradation and fermentation, maintaining energy metabolism, and improving nutrient utilization [34]. Therefore, changes in the composition and function of the rumen microbial flora are of great significance for understanding how microbial communities interact with host phenotypes [35]. Yi Simeng et al. [36] showed that at the phylum level in rumen fluid, *Bacteroidota* and *Firmicutes* were the dominant phyla, which is consistent with the results of this experiment, where *Bacteroidota* and *Firmicutes* were identified as dominant phyla in rams fed HMC. However, between the two groups, the abundance of *Firmicutes_A* was significantly higher in the GS group than in the CT group, consistent with the results of Gao Y [37] who fed different levels of HMC to dairy cows and found a significant increase in the relative abundance of *Firmicutes* in rumen fluid. In the digestive system of ruminants, *Firmicutes* and *Bacteroidota* play important roles [38]. *Bacteroidota* are mainly involved in the decomposition of non-fibrous substances and plant polysaccharides, while *Firmicutes* are primarily responsible for the digestion of dietary fiber [39]. These microbial groups can secrete specific enzymes to break down dietary fiber, proteins, and digestible carbohydrates ingested by the host, assisting the host in completing metabolic processes, generating metabolic and net energy, which is crucial for maintaining host health and energy supply [40].

At the genus level, *Prevotella*, *Cryptobacteroides*, and *Succiniclasticum* were the dominant taxa in this study. As core members of the rumen microbial community, these genera play a key role in maintaining the balance between animal health and disease [41]. *Cryptobacteroides*, which belong to the family Bacteroidaceae, currently lack a pure culture isolate and remains classified as a candidate genus (Candidatus). Due to its high phylogenetic similarity to the genus Bacteroides, it is also referred to as “cryptic Bacteroides.” Notably, unlike traditional members of the Bacteroides genus, *Cryptobacteroides* is Gram-positive, a cell wall structural feature that may confer greater tolerance to specific environmental stresses. Functional genomic studies indicate that *Cryptobacteroides* play an important role in the metabolism of methyl-containing compounds [42]. The density of genes encoding carbohydrate-active enzymes (CAZymes) in its genome is significantly higher than that of other Bacteroidota members, enabling it to efficiently degrade plant fibers and convert the metabolites into short-chain fatty acids, thereby providing energy to the host [43]. In the present study, the abundance of *Cryptobacteroides* was significantly higher in the GS group than in the CT group. This finding suggests that feeding HMC may allow *Cryptobacteroides* to further degrade fibrous substrates via its efficient CAZymes system, producing substantial amounts of VFA and enabling the experimental sheep to obtain more energy directly from rumen fermentation products. In conclusion, feeding HMC significantly influences the dominant microbial community structure at the genus level in the rumen of Kazakh rams. Specifically, by enriching functional taxa such as *Cryptobacteroides*, it optimizes the rumen fermentation pattern and improves energy utilization efficiency, playing an important role in maintaining the health and rumen homeostasis of Kazakh rams.

### 4.4. Effects of Feeding HMC on Rumen Metabolomics in Kazakh Rams

To elucidate the potential mechanisms by which HMC influences the rumen environment and host physiology, this study employed metabolomics to profile rumen fluid from Kazakh rams. Orthogonal partial least squares discriminant analysis (OPLS-DA) revealed a clear separation in metabolic profiles between the CT and GS groups. Notably, the differential metabolites identified were significantly enriched in the GSH pathway, suggesting that HMC feeding modulates the rumen redox environment.

Spermidine is a polyamine involved in cellular redox regulation and immune function. Its concentration is closely associated with oxidative stress and inflammatory response [44,45]. In the present study, the observed decrease in spermidine in the GS group may reflect a reduced oxidative challenge and contribute to the improved immune status in HMC-fed animals.

1,5-Diaminopentane, also known as cadaverine, is a product of lysine decarboxylation, primarily generated by microbial lysine decarboxylase. In ruminants, cadaverine accumulation in the rumen is often associated with excessive protein degradation or imbalanced microbial fermentation. This is often the way microorganisms handle excess amino acids in acidic or stressful environments [46]. Studies have shown that odors in dairy cow barns mainly come from harmful substances such as ammonia, cadaverine, and phenol, which are products of protein decomposition by Escherichia coli [47]. Acidic environments can increase cadaverine production. In the rumen environment, elevated levels of cadaverine may reflect excessive proteolysis or microbial metabolic stress. The reduced cadaverine concentration observed in the GS group suggests that HMC feeding may alleviate protein fermentation pressure and contribute to a more stable rumen microbial ecosystem.

5-Oxoproline, an intermediate in the γ-glutamyl cycle, is a sensitive indicator of GSH turnover and antioxidant demand [48]. The significant increase in 5-oxoproline in the GS group aligns with the activation of the GSH pathway. This finding is consistent with the elevated serum activities of SOD and CAT observed in these animals, indicating a systemic enhancement of antioxidant capacity. Similarly, the dynamic balance between dehydroascorbic acid and its reduced form, ascorbic acid (vitamin C), reflects the cellular redox state. Vitamin C is not only a critical antioxidant but also plays an essential role in modulating immune cell function and regulating inflammatory responses [49,50]. The altered abundance of these metabolites collectively points to an improved redox balance and enhanced immune preparedness in rams consuming HMC.

In summary, rumen metabolomic analysis revealed that feeding HMC to Kazakh rams significantly altered the ruminal metabolic profile, with differential metabolites primarily enriched in the glutathione metabolism pathway. The observed increase in 5-oxoproline and dehydroascorbic acid, coupled with decreased spermidine and cadaverine, indicates enhanced antioxidant capacity within the rumen environment. These metabolic changes align with the elevated serum activities of SOD and CAT, suggesting a coordinated improvement in systemic redox homeostasis. Furthermore, the activation of glutathione metabolism provides a mechanistic link between HMC feeding and the enhanced immune function observed through increased serum IgA and IgM levels. These findings demonstrate that HMC not only serves as an energy-dense feed but also actively modulates ruminal metabolic pathways to improve host antioxidant capacity, immune function, and metabolic homeostasis.

## 5. Conclusions

Feeding HMC significantly improved feed conversion efficiency in Kazakh rams while enhancing immune function and antioxidant capacity. No significant changes were observed in the α-diversity of rumen microbiota; however, the relative abundance of Firmicutes_A at the phylum level and Cryptobacteroides at the genus level increased significantly. Rumen metabolomics analysis revealed differential metabolites significantly enriched in the glutathione metabolism pathway. These results suggest that HMC may regulate host metabolism and physiological status by reshaping the structure and metabolic functions of dominant rumen microbiota, though the specific mechanisms require further elucidation.

This study provides a theoretical foundation for a deeper understanding of the microbial and metabolic mechanisms regulating ruminant health in HMC. On this basis, further revealing the interaction between key microbial communities and core metabolites will contribute to the development of precision nutrition strategies based on rumen microecology regulation, offering important technical support for improving the quality and efficiency of meat sheep farming in Xinjiang and northern agricultural regions of China, as well as achieving green and sustainable development.

## Figures and Tables

**Table 1:** Feed formulation and nutrient level of basic diet (DM %).

Items	Treatment ^(1)^	
	CT	GS
Concentrate ingredients,% ^(2)^		
Whole plant corn silage	30	30
Cotton residu	30	30
Ordinary crushed corn	24	12
High-moisture corn	0	12
Cottonseed meal	5	5
Soybean meal	5	5
Wheat bran	3.2	3.2
Premix ^(3)^	2	2
NaCl	0.4	0.4
NaHCO_3_	0.4	0.4
Total	100	100
Nutritional level ^(4)^		
Gross Energy GE (MJ/kg)	15.81	15.83
Crude protein CP (%)	13.05	13.07
Crude fat EE (%)	4.21	4.34
Acid Detergent Fiber ADF (%)	25.35	26.19
Neutral Detergent Fiber NDF (%)	67.25	68.01
Ash (%)	8.35	8.64
Calcium Ca (%)	0.66	0.67
Phosphorus P (%)	0.34	0.35

^(1)^ CT: control group (diet with only ordinary crushed corn); GS: experimental group (diet with 50% ordinary crushed corn + 50% HMC). ^(2)^ All feed ingredients (whole plant corn silage, cotton residue, ordinary crushed corn, high-moisture corn, cottonseed meal, soybean meal, wheat bran) were purchased from Xinjiang Tianrun Dairy Co., Ltd. (Urumqi, China). ^(3)^ The premix provided per kilogram of diet contained: vitamin A, 130,000–250,000 IU; vitamin D_3_, 40,000–100,000 IU; vitamin E, ≥1400 IU; copper (from tribasic copper chloride), 550–800 mg; iron (from ferrous sulfate), 1500–7000 mg; manganese (from manganese sulfate), 1100–3000 mg; zinc (from zinc sulfate), 800–2000 mg; iodine (from calcium iodate), 20–30 mg; selenium, 8–12 mg; cobalt, 20–30 mg; calcium, 10–20%; total phosphorus, 1.5%; sodium chloride, 12–20%; and moisture, ≤10.0%. All nutrient composition values are calculated values. ^(4)^ All nutrient levels are presented on a dry matter basis, nutritional levels are measured values [12].

**Table 2:** Nutritional components of ordinary crushed corn and HMC (DM%).

Nutrient Component	Ordinary Crushed Corn	HMC
Crude protein CP	8.12	8.45
Crude fat EE	4.13	4.32
Neutral Detergent Fiber NDF	22.15	20.60
Acid Detergent Fiber ADF	7.72	6.14

**Table 3:** Effects of feeding HMC on weight performance of Kazakh rams.

Items	Treatment ^(1)^		*p*-Value
	CT	GS	
IBW/kg ^(2)^	35.19 ± 4.68	35.81 ± 3.27	0.27
FBW/kg ^(3)^	59.23 ± 9.21	61.94 ± 9.99	0.97
Net weight gain/kg	24.04 ± 5.28	26.13 ± 6.84	0.17
ADG (kg/d) ^(4)^	0.20 ± 0.04	0.22 ± 0.07	0.17
ADFI (kg/d) ^(5)^	2.28 ± 0.49	2.14 ± 0.04	0.09
F/G ^(6)^	8.55 ± 0.09 ^a^	7.29 ± 0.17 ^b^	0.01

^(1)^ CT, control group (diet with ordinary crushed corn); GS, experimental group (diet with 50% ordinary crushed corn + 50% HMC); ^(2)^ IBW, initial body weight; ^(3)^ FBW, final body weight; ^(4)^ ADG, average daily gain; ^(5)^ ADFI, average daily feed intake; ^(6)^ F/G, feed-to-gain ratio. Values are presented as mean ± standard deviation. Different lowercase superscript letters within the same row indicate significant difference (*p* < 0.05).

**Table 4:** Effects of feeding HMC on serum immune indices of Kazakh rams.

Items	Times	Treatment		*p*-Value
		CT	GS	
IgA (g/L) ^(1)^	0 d	0.93 ± 0.11	1.04 ± 0.06	0.11
	40 d	0.93 ± 0.11 ^B^	1.18 ± 0.05 ^A^	<0.01
	80 d	1.20 ± 0.11	1.25 ± 0.05	0.79
	120 d	1.18 ± 0.19	1.28 ± 0.05	0.28
IgG (g/L) ^(2)^	0 d	15.75 ± 2.03	16.53 ± 1.05	0.42
	40 d	16.12 ± 2.57	17.45 ± 1.65	0.31
	80 d	16.99 ± 3.06	17.22 ± 0.85	0.87
	120 d	17.42 ± 2.35	17.93 ± 0.38	0.62
IgM (g/L) ^(3)^	0 d	0.58 ± 0.11	0.67 ± 0.03	0.12
	40 d	0.58 ± 0.09 ^b^	0.69 ± 0.03 ^a^	0.03
	80 d	0.64 ± 0.07 ^b^	0.72 ± 0.02 ^a^	0.04
	120 d	0.67 ± 0.05 ^b^	0.74 ± 0.03 ^a^	0.02
IL-1β (pg/mL) ^(4)^	0 d	31.24 ± 6.71	31.84 ± 4.31	0.86
	40 d	25.40 ± 2.21	26.15 ± 2.17	0.54
	80 d	24.05 ± 2.51	24.81 ± 0.59	0.58
	120 d	22.58 ± 1.79	23.27 ± 1.78	0.66
TNF-α (pg/mL) ^(5)^	0 d	67.18 ± 12.53	69.61 ± 2.36	0.72
	40 d	62.23 ± 14.52	63.50 ± 7.51	0.85
	80 d	53.44 ± 6.74	55.34 ± 6.82	0.71
	120 d	49.45 ± 5.98	48.71 ± 1.49	0.85

^(1)^ IgA, immunoglobulin A; ^(2)^ IgG, immunoglobulin G; ^(3)^ IgM, immunoglobulin M; ^(4)^ IL-1β, interleukin-1β; ^(5)^ TNF-α, tumor necrosis factor-α. Values are presented as mean ± standard deviation. Different lowercase superscript letters within the same row indicate significant difference (*p* < 0.05), while different uppercase superscript letters indicate highly significant difference (*p* < 0.01).

**Table 5:** Effects of feeding HMC on serum antioxidant indices of Kazakh rams.

Items	Times	Treatment		*p*-Value
		CT	GS	
SOD (U/mL) ^(1)^	0 d	53.36 ± 12.32	50.54 ± 6.54	0.63
	40 d	71.20 ± 13.18	71.06 ± 7.70	0.98
	80 d	66.13 ± 13.67	83.69 ± 14.11	0.05
	120 d	71.74 ± 9.68 ^B^	89.89 ± 5.01 ^A^	<0.01
MDA (nmol/mL) ^(2)^	0 d	4.94 ± 0.51	4.70 ± 0.62	0.49
	40 d	4.38 ± 0.68	4.13 ± 0.62	0.53
	80 d	4.08 ± 0.67	3.79 ± 0.48	0.4
	120 d	3.71 ± 0.56	3.31 ± 0.37	0.18
CAT (U/mL) ^(3)^	0 d	30.40 ± 3.30	34.37 ± 3.03	0.06
	40 d	31.94 ± 4.59 ^B^	39.14 ± 2.83 ^A^	<0.01
	80 d	31.21 ± 5.22 ^B^	43.74 ± 4.55 ^A^	<0.01
	120 d	31.90 ± 4.74 ^B^	47.71 ± 4.14 ^A^	<0.01
T-AOC (U/mL) ^(4)^	0 d	11.84 ± 2.61	9.19 ± 0.15	0.09
	40 d	9.44 ± 2.16	9.76 ± 0.86	0.82
	80 d	11.51 ± 3.11	9.29 ± 0.19	0.29
	120 d	9.14 ± 2.04	9.31 ± 0.39	0.85

^(1)^ SOD, superoxide dismutase; ^(2)^ MDA, malondialdehyde; ^(3)^ CAT, catalase; ^(4)^ T-AOC, total antioxidant capacity. Values are presented as mean ± standard deviation. Different uppercase superscript letters within the same row indicate significant difference (*p* < 0.01).

**Table 6:** Effects of HMC on rumen fluid fermentation in Kazakh rams.

Items	Treatment		*p*-Value
	CT	GS	
pH ^(1)^	6.39 ± 0.21	6.76 ± 0.17	0.07
Acetic (mmol/L)	69.54 ± 7.33	61.04 ± 4.54	0.16
Propioni (mmol/L)	23.49 ± 2.89	19.56 ± 1.15	0.09
Butyric (mmol/L)	21.51 ± 0.55 ^A^	16.22 ± 1.21 ^B^	<0.01
Isobutyric (mmol/L)	0.79 ± 0.08	1.16 ± 0.27	0.09
2-Methylbutyrate (mmol/L)	0.39 ± 0.05 ^b^	0.88 ± 0.24 ^a^	0.03
Valeric (mmol/L)	1.52 ± 0.13 ^b^	1.85 ± 0.11 ^a^	0.03
Isovaleric (mmol/L)	0.71 ± 0.07	1.01 ± 0.19	0.06
Hexanoic acid (mmol/L)	1.14 ± 0.11	1.42 ± 0.22	0.13
Acetic Acid/Propionic Acid Ratio	2.96 ± 2.54	3.12 ± 3.96	0.18
TVFA ^(2)^	117.56 ± 11.05	100.84 ± 7.74	0.26

^(1)^ pH indicates the acidity/alkalinity of rumen fluid 3 h after feeding; ^(2)^ TVFA, Total volatile fatty acid. Values are presented as mean ± standard deviation. Different lowercase superscript letters within the same row indicate significant difference (*p* < 0.05), while different uppercase superscript letters indicate highly significant difference (*p* < 0.01).

**Table 7:** Rumen differential metabolic pathways and their differential metabolites.

Metabolic Pathway	*p*-Value	Differential Metabolites	VIP	*p*-Value
Glutathione metabolism	0.03	Spermidine ↓	1.48	<0.01
		Cadaverine ↓	1.01	0.01
		Dehydroascorbic acid ↑	1.24	<0.01
		5-oxoproline ↑	1.29	<0.01
Beta-Alanine metabolism	0.08	Vitamin B5 ↑	1.05	<0.01
		Anserine ↓	1.27	<0.01
		Spermidine ↓	1.49	<0.01
Sphingolipid metabolism	0.08	Sphinganine ↓	1.06	<0.01
		Phosphorylethanolamine ↑	1.17	<0.01
		Psychosine ↑	1.08	<0.01
Lysine degradation	0.09	deoxycarnitine ↑	1.01	<0.01
		L-Saccharopine ↑	1.41	<0.01
		L-A-HYDROXYGLUTARIC ACID DISODIUM) ↑	1.14	<0.01
		Cadaverine ↓	1.01	0.01
		L-Pipecolic acid ↑	1.21	<0.01

The screening of differential metabolites primarily references three parameters: VIP, FC, and *p*-value. VIP refers to the Variable Importance in the Projection of the first principal component in the PLS-DA model, indicating the contribution of metabolites to grouping. FC denotes the fold change, calculated as the ratio of the mean quantitative value of each metabolite across all biological replicates in the comparison group. The *p*-value is derived from T-tests and represents the significance level of the difference. The threshold is set as VIP > 1.0, FC > 1.5, or FC < 0.667 with *p*-value < 0.05.

## Data Availability

The data presented in this study are available on request from the corresponding author.

## Abbreviations

The following abbreviations are used in this manuscript:

HMC	High Moisture Corn
CT	Control Group (diet with only ordinary crushed corn)
GS	Experimental Group (diet with 50% ordinary crushed corn + 50% HMC)
GE	Gross Energy
Ca	Calcium
P	Phosphorus
EE	Ether Extract
ADF	Acid Detergent Fiber
NDF	Neutral Detergent Fiber
IBW	Initial Body Weight
FBW	Final Body Weight
ADG	Average Daily Gain
ADFI	Average Daily Feed Intake
DM	Dry Matter
F/G	Feed-to-Gain Ratio
IgA	Immunoglobulin A
IgG	Immunoglobulin G
IgM	Immunoglobulin M
IL-1β	Interleukin-1β
TNF-α	Tumor Necrosis Factor-α
SOD	Superoxide Dismutase
MDA	Malondialdehyde
CAT	Catalase
T-AOC	Total Antioxidant Capacity
VFA	Volatile Fatty Acid
KEGG	Kyoto Encyclopedia of Genes and Genomes
GSH	Glutathione metabolism
γ-GCT	γ-glutamyl transpeptidase
Vc	vitamin C
CAZymes	carbohydrate-active enzymes

## References

[1] Elmhadi M.E., Ali D.K., Khogali M.K., Wang H. (2022). Subacute ruminal acidosis in dairy herds: Microbiological and nutritional causes, consequences, and prevention strategies. Anim. Nutr..

[2] Coulson C.A., Boyd B.M., Troyer B.C., McPhillips L.J., Norman M.M., Woita N.M., Wilson H.C., Butterfield K.M., Spore T.J., Erickson G.E. (2023). Impact of different corn milling methods for high-moisture and dry corn on finishing cattle performance, carcass characteristics, and nutrient digestion. J. Anim. Sci..

[3] Ferraretto L.F., Crump P.M., Shaver R.D. (2013). Effect of cereal grain type and corn grain harvesting and processing methods on intake, digestion, and milk production by dairy cows through a meta-analysis. J. Dairy Sci..

[4] Wu Z., Luo Y., Bao J., Luo Y., Yu Z. (2020). Additives affect the distribution of metabolic profile, microbial communities and antibiotic resistance genes in high-moisture sweet corn kernel silage. Bioresour. Technol..

[5] Guo K.J., Shang S.L., Li J.J., Dou J.H. (2022). Progress of high moisture corn process technique and its application. Chin. J. Anim. Nutr..

[6] Han J., Song L., Zhang C., Zhang J., Liu K. (2018). Effects of feeding high-moisture ensiled corn on production performance and feeding cost of dairy cows. Feed Ind..

[7] Zhang H.W., Yang X.Y., Yu B., Ren D.X., Du H.R., Lin S., Guo J.J. (2024). Progress in making high moisture wet storage corn and its application in dairy (meat) cattle production. Cereal Feed Ind..

[8] Carey R.E., Paddock Z.D., Ribeiro G.O., McAllister T.A., Penner G.B. (2023). Digestibility of western Canadian finishing beef cattle diets when short-season, high-moisture shelled corn and snaplage partially replace dry-rolled barley grain and barley silage. Can. J. Anim. Sci..

[9] Purwin C., Opyd P.M., Baranowska M., Borsuk-Stanulewicz M. (2022). The effect of diets containing high-moisture corn or triticale grain on animal performance and the fatty acid composition of lamb muscles. Animals.

[10] Li Z.Q. (2020). Study on the Growth and Development Patterns of Kazakh Sheep and Their Crossbred Progeny. Master’s Thesis.

[11] Qi G. (2016). Nutritional requirements of meat sheep. Mod. Anim. Husb. Sci. Technol..

[12] Lebret B., Lhuisset S., Labussière E., Louveau I. (2023). Combining pig genetic and feeding strategies improves the sensory, nutritional and technological quality of pork in the context of relocation of feed resources. Meat Sci..

[13] (2008). Laboratories—General Requirements for Biosafety.

[14] Krupová Z. (2020). Economic weights of current and new breeding objective traits in Aberdeen Angus. Czech J. Anim. Sci..

[15] Stock R.A., Sindt M.H., Cleale R.M., Britton R.A. (1991). High-moisture corn utilization in finishing cattle. J. Anim. Sci..

[16] Hopfauf S.M., Boyd B.M., McPhillips L.J., Erickson G.E. (2020). Impact of feeding *Aspergillus subspecies* (ssp.) blend and different corn processing methods on finishing beef cattle performance and carcass characteristics. J. Anim. Sci..

[17] Fan Y., Li S.L., Kong F.L., Wang W. (2022). Influencing factors of nutritional value of high moisture corn and its application in dairy production. Chin. J. Anim. Nutr..

[18] Tang F., Li J.A., Dai G.C., Ou D.Y., Xiong F.L., Yao H.Y. (2023). Effects of chicory polysaccharide on growth performance, immune function, and intestinal microbiota in immunosuppressed broilers. Chin. J. Anim. Sci..

[19] Wang J.Q., Yin F.G., Zhu C., Yu H., Niven S.J., de Lange C.F.M., Gong J. (2012). Evaluation of probiotic bacteria for their effects on the growth performance and intestinal microbiota of newly-weaned pigs fed fermented high-moisture maize. Livest. Sci..

[20] Wang J.F., Yang G.Y., Fan Z.H., Liu Q., Zhang P.C., Ren Y.S., Zhang C.X. (2021). Effects of whole plant corn silage ratio in diet on growth performance, rumen fermentation, nutrient digestibility and serological parameters of Dorper×Hu crossbred female lambs. Sci. Agric. Sin..

[21] Sharma N., Ranjitkar S., Sharma N.K., Engberg R.M. (2017). Influence of feeding crimped kernel maize silage on the course of subclinical necrotic enteritis in a broiler disease model. Anim. Nutr..

[22] Pickworth C.L., Loerch S.C., Kopec R.E., Schwartz S.J., Fluharty F.L. (2012). Concentration of pro-vitamin A carotenoids in common beef cattle feedstuffs. J. Anim. Sci..

[23] Jin L., Yan S., Shi B., Bao H., Gong J., Guo X., Li J. (2014). Effects of vitamin A on the milk performance, antioxidant functions and immune functions of dairy cows. Anim. Feed Sci. Technol..

[24] Sakamoto T., Imai H. (2017). Hydrogen peroxide produced by superoxide dismutase SOD-2 activates sperm in Caenorhabditis elegans. J. Biol. Chem..

[25] Shang S.L., Li T., Li J.J., Hu T.T., Chen Y.D., Li D., Liu G.F., Guo K.J. (2023). Effects of high moisture ear corn on performance, serum biochemical indices, immune function and antioxidant capacity of dairy cows. Chin. J. Anim. Nutr..

[26] Fang Z., Xue B., Liu L.Z., Yang Y. (2015). Free radical types in cells and generation mechanism. J. Anhui Agric. Sci..

[27] Gao D., Gao Z., Zhu G. (2013). Antioxidant effects of Lactobacillus plantarum via activation of transcription factor Nrf2. Food Funct..

[28] Plaizier J.C., Li S., Danscher A.M., Derakshani H., Andersen P.H., Khafipour E. (2017). Changes in microbiota in rumen digesta and feces due to a grain-based subacute ruminal acidosis (SARA) challenge. Microb. Ecol..

[29] Peng S.Y., Qu Y.L., Gao W.X., Qu J.C., Liu H.Z., Miao R.F. (2022). Effects of different combination ratios of ordinary crushed corn and wet-stored corn on rumen in vitro fermentation parameters in dairy cows. Chin. J. Anim. Sci..

[30] Knowlton K.F., Glenn B.P., Erdman R.A. (1998). Performance, ruminal fermentation, and site of starch digestion in early lactation cows fed corn grain harvested and processed differently. J. Dairy Sci..

[31] Bergman E.N. (1990). Energy contributions of volatile fatty acids from the gastrointestinal tract in various species. Physiol. Rev..

[32] Xie B., Zhang N.F., Zhang C.X., Diao Q.Y. (2018). Effects of roughage on rumen development in young ruminants and its mechanism. Chin. J. Anim. Nutr..

[33] Bach A., Joulie I., Chevaux E., Elcoso G., Ragués J. (2021). Milk performance and rumen microbiome of dairy cows as affected by the inclusion of corn silage or corn shredlage in a total mixed ration. Animal.

[34] Arowolo M.A., He J. (2018). Use of probiotics and botanical extracts to improve ruminant production in the tropics: A review. Anim. Nutr..

[35] Lavelle A., Sokol H. (2020). Gut microbiota-derived metabolites as key actors in inflammatory bowel disease. Nat. Rev. Gastroenterol. Hepatol..

[36] Yi S., Dai D., Wu H., Chai S., Liu S., Meng Q., Zhou Z. (2022). Dietary concentrate-to-forage ratio affects rumen bacterial community composition and metabolome of yaks. Front. Nutr..

[37] Gao Y. (2025). Effects of Feeding High-Moisture Corn on Growth, Serum Biochemistry, and Rumen Metabolism in Young Simmental-Crossbred Bulls. Master’s Thesis.

[38] Jami E., Israel A., Kotser A., Mizrahi I. (2013). Exploring the bovine rumen bacterial community from birth to adulthood. ISME J..

[39] Evans N.J., Brown J.M., Murray R.D., Getty B., Birtles R.J., Hart C.A., Carter S.D. (2011). Characterization of novel bovine gastrointestinal tract Treponema isolates and comparison with bovine digital dermatitis treponemes. Appl. Environ. Microbiol..

[40] Ukhanova M., Culpepper T., Baer D., Gordon D., Kanahori S., Valentine J., Neu J., Sun Y., Wang X., Mai V. (2012). Gut microbiota correlates with energy gain from dietary fibre and appears to be associated with acute and chronic intestinal diseases. Clin. Microbiol. Infect..

[41] Tett A., Pasolli E., Masetti G., Ercolini D., Segata N. (2021). Prevotella diversity, niches and interactions with the human host. Nat. Rev. Microbiol..

[42] Deng Y.C. (2024). Effects of Different Proportions of Brewer’s Grains on Lactation Performance, Nutrient Digestibility and Rumen Microorganisms in Dairy Buffaloes. Master’s Thesis.

[43] Northwest Plateau Institute of Biology (2024). New Breakthroughs in the Genetic Evolution of Gut Microbiota in Ungulates on the Northwest Plateau. http://lzb.cas.cn/kyjz/202411/t20241107_7435559.html.

[44] Uemura T., Matsunaga M., Yokota Y., Takao K., Furuchi T. (2023). Inhibition of polyamine catabolism reduces cellular senescence. Int. J. Mol. Sci..

[45] Ivanova O.N., Gavlina A.V., Karpenko I.L., Zenov M.A., Antseva S.S., Zakirova N.F., Elliston V., Krasnov G.S., Fedyakina I.T., Vorobyev P.O. (2024). Polyamine catabolism revisited: Acetylpolyamine oxidase plays a minor role due to low expression. Cells.

[46] Kind S., Wittmann C. (2011). Bio-based production of the platform chemical 1,5-diaminopentane. Appl. Microbiol. Biotechnol..

[47] Song Y.H., Hu W.J. (2008). Application of microbial ecological agent in dairy cows. J. Xichang Coll. (Nat. Sci. Ed.).

[48] Bachhawat A.K., Yadav S. (2018). The glutathione cycle: Glutathione metabolism beyond the γ-glutamyl cycle. IUBMB Life.

[49] Bai L., Xie Z.L., Wang Z.W., Hu L.Y., He Y.K., Wu G.Q. (2021). Therapeutic effect of vitamin C on inflammation. J. Clin. Pathol. Res..

[50] Yang X.D. (2026). Study on the application of scientific feeding management and breeding techniques and the optimization of disease prevention and control measures in sheep. Friends Farmers Get Rich.

